# Overcoming Microstructural Defects at the Buried Interface of Formamidinium-Based Perovskite Solar Cells

**DOI:** 10.1021/acsami.4c11052

**Published:** 2024-08-27

**Authors:** Heng-Yi Lin, Zhongyao Jiang, Shi-Chun Liu, Zhaoyi Du, Shih-En Hsu, Yun-Shan Li, Wei-Jia Qiu, Hongta Yang, Thomas J. Macdonald, Martyn A. McLachlan, Chieh-Ting Lin

**Affiliations:** † Department of Chemical Engineering, National Chung Hsing University, 145 Xingda Road, Taichung 40227, Taiwan; ‡ Department of Materials, Molecular Sciences Research Hub, Imperial College London, 82 Wood Ln, London W12 0BZ, U.K.; § Department of Electronic & Electrical Engineering, University College London, Torrington Place, London WC1E 7JE, U.K.; ∥ Innovation and Development Center of Sustainable Agriculture, National Chung Hsing University, 145 Xingda Road, Taichung 40227, Taiwan

**Keywords:** perovskite solar cells, buried interface, device photoluminescence, charge extraction, microstructural defects, methylammonium chloride, wide processing window

## Abstract

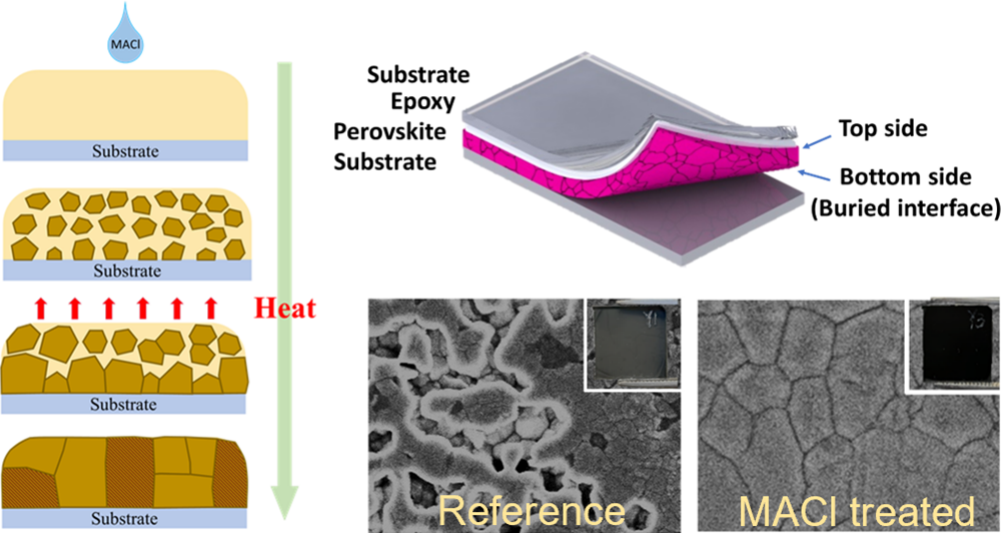

Since the advent of formamidinium (FA)-based perovskite photovoltaics (PVs), significant performance enhancements have been achieved. However, a critical challenge persists: the propensity for void formation in the perovskite film at the buried perovskite–interlayer interface has a deleterious effect on device performance. With most emerging perovskite PVs adopting the p-i-n architecture, the specific challenge lies at the perovskite–hole transport layer (HTL) interface, with previous strategies to overcome this limitation being limited to specific perovskite–HTL combinations; thus, the lack of universal approaches represents a bottleneck. Here, we present a novel strategy that overcomes the formation of such voids (microstructural defects) through a film treatment with methylammonium chloride (MACl). Specifically, our work introduces MACl via a sequential deposition method, having a profound impact on the microstructural defect density at the critical buried interface. Our technique is independent of both the HTL and the perovskite film thickness, highlighting the universal nature of this approach. By employing device photoluminescence measurements and conductive atomic force microscopy, we reveal that when present, such voids impede charge extraction, thereby diminishing device short-circuit current. Through comprehensive steady-state and transient photoluminescence spectroscopy analysis, we demonstrate that by implementing our MACl treatment to remedy these voids, devices with reduced defect states, suppressed nonradiative recombination, and extended carrier lifetimes of up to 2.3 μs can be prepared. Furthermore, our novel treatment reduces the stringent constraints around antisolvent choice and dripping time, significantly extending the processing window for the perovskite absorber layer and offering significantly greater flexibility for device fabrication.

## Introduction

Organic–inorganic lead halide perovskite solar cells (PSCs) have gained significant attention over the past decade due to their remarkable optoelectronic properties, with the highest certified power conversion efficiency (PCE) reaching 26.1%.^1−3^ Among the diverse variants of perovskites, formamidinium lead iodide (FAPbI_3_) has emerged as a favorite due to its near-ideal bandgap, aligning closely with the Shockley–Queisser limit.^4−6^ However, FAPbI_3_ is known for its phase instability, existing as a black, photoactive α-phase, which is optimal for solar cell applications, and the less desirable photoinactive yellow δ-phase.^4−8^ Several strategies have been developed to increase the stability of the α-phase, perhaps the most common being compositional engineering, whereby small cations, e.g., methylammonium (MA) and cesium (Cs) or anions, e.g., bromide and chloride^9,10^ are incorporated into the structure. In some cases, larger cations, e.g., phenethylammonium iodide (PEAI) have also been incorporated for phase stabilization.^11^ However, such strategies can result in changes in the bandgap of FAPbI_3_ and may also result in phase segregation.^12^ To overcome such unintentional modifications to the optical and microstructural properties of FAPbI_3_, a number of methods have been proposed that aim to improve stability and enhance performance, including postdeposition aerosol treatment and annealing,^5,13^ and controlling the composition and stoichiometry of adjacent device interlayers.^14^

Interestingly, a large volume of the research focusing on improving PSC performance largely focuses on the structure and properties of the upper surface of the perovskite absorber, i.e., the perovskite–air interface, with subsurface analysis typically being achieved by cross-sectional imaging of the active layer and complete devices.^15−21^ This is attributed to the ease of access to the top surface for the purposes of imaging and the evaluation of compatible interlayer materials through solution and thermal deposition routes. The work of Yang et al. demonstrated a direct link between inhomogeneities at the less studied buried interface and associated nonradiative losses.^22^ Their innovative use of a facile lift-off method to directly expose the buried interface underscores its importance and opens new avenues for optimizing PSC efficiency by focusing on this interface, yet it is intriguing that this buried interface is less explored in the literature, despite the critical role it plays in device performance.^22−25^ Most strategies aimed at reducing losses that occur at the buried interface and enhancing PCE are typically achieved through modifying the adjacent charge transport layer—for example, the use of lattice-matched SrSnO_3_ as an electron transport layer (ETL) in n-i-p PSCs, creating periodic halide perovskite crystal lattices with low defect densities at the buried interface.^24^ In p-i-n architectures, poly(triarylamine) (PTAA) is commonly employed as a hole transport layer (HTL) despite being inherently hydrophobic—a property that manifests in the formation of defects at the buried interface.^25,26^ To address this, tetrachloroaluminate anions (AlCl4^–^) have been doped into PTAA to improve surface wettability and reduce defects, and the results are enhanced hole extraction and transport and a reduction of nonradiative recombination at the HTL/perovskite interface.^27^ Introducing a 2D perovskite layer at the buried interface reduces interfacial nonradiative recombination, resulting in higher open-circuit voltage (*V*_OC_) in PSCs,^28^ driven by modifications of crystallinity and band alignment of the perovskite film at the buried interface. Finally, the incorporation of small molecules at the buried interface can result in a number of improvements, e.g., 2-hydroxy-4-methoxybenzophenone-5-sulfonic acid (HMBS), which acts as a cross-linker that reduces surface trap states in the ETL, optimizing energy level alignment at the buried ETL/perovskite interface, and enhances crystallinity of the perovskite.^23^ Despite the improvements offered by such strategies, they are limited to specific materials and specific device architectures. A general approach that can be applied to a variety of devices and interlayers has not yet been proposed.

Here, using p-i-n architectures, we investigate the density of microstructural defects present at the buried interface and correlate their presence with perovskite active layer thickness using a range of common HTLs. Using a combination of conductive atomic force microscopy (C-AFM) and photoluminescence quenching (PLQ) in complete devices under open- and short-circuit conditions, we demonstrate that these microstructural defects, observed as voids in perovskite films, hinder charge transport to the external circuit and adversely affect device short-circuit current density (*J*_SC_). We consequently demonstrate a facile strategy based on sequential deposition post-treatment that can significantly improve the quality of this buried interface. We note recent work reporting the incorporation of methylammonium chloride (MACl) through additive engineering in FA-based perovskites^6,10^ or postannealing solvent vapor treatment in MAPbI_3_^29^ and highlight the novelty of our sequential deposition method and the profound impact it has on eliminating microstructural defects at buried interfaces. We demonstrate the applicability of our methodology using a range of perovskite active layer thicknesses, correlating this with defect density, and through the use of a range of HTLs. Additionally, we demonstrate that our methodology expands the current processing window for perovskite active layers, reducing susceptibility to variations in the type and dripping time of the antisolvent. Specifically, our approach is independent of perovskite film thickness, thereby opening future avenues for a diverse range of applications beyond photovoltaics (PVs). This includes areas such as X-ray detectors, which typically require thick (>1 μm) perovskite films.^30,31^

## Results and Discussion

### Uncovering Voids Located at the Substrate Interface of Perovskite Thin Films

In all the data presented, we have utilized FA_0.97_MA_0.03_Pb(I_0.97_Br_0.03_)_3_ as the perovskite layer; this specific composition minimizes possible phase transitions that may occur during preparation and characterization.^32^ This is particularly important given the known phase instability of FAPbI_3_ at room temperature, where it can switch from a black photoactive α-phase to a yellow photoinactive δ-phase.^4,5^ For the initial work, we rely on MeO-2PACz, a well-studied self-assembled monolayer (SAM), as the device HTL owing to its excellent processability, compatibility with our perovskite, and intrinsic charge transport properties.^33,34^

**Figure 1:**
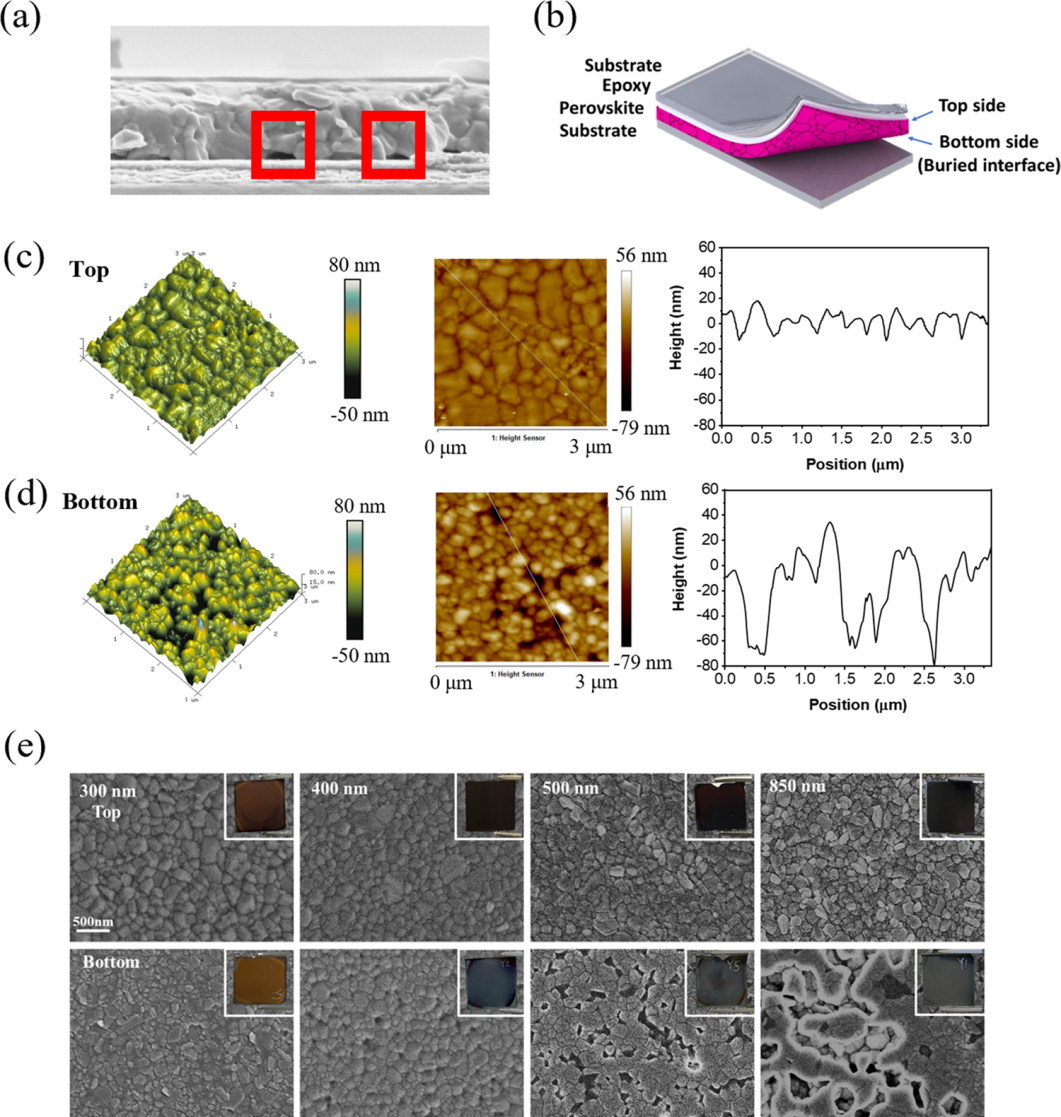
(a) Cross-section SEM image of a 400 nm FA_0.97_MA_0.03_Pb(I_0.97_Br_0.03_)_3_ film, red squares superimposed to highlight the voids at the buried interface. (b) Schematic showing epoxy delamination methodology. AFM images of (c) top and (d) bottom surfaces of the films. (e) Top-view and bottom-view SEM images of perovskite films over the thickness range of 200–850 nm. The inset photographs show the appearance of the films when viewed from the top and bottom.

A typical cross-sectional scanning electron microscope (SEM) image of a 400 nm thick FA_0.97_MA_0.03_Pb(I_0.97_Br_0.03_)_3_ film processed on MeO-2PACz is shown in Figure 1a. The anticipated polycrystalline structure of the perovskite is observed, and the top surface of the film appears relatively flat and homogeneous. The superimposed red squares highlight the presence of large voids at the HTL–perovskite buried interface; such voids have been previously reported and are known to be detrimental to the device performance.^22,25^ To explore the buried interface, we employed an epoxy delamination^32^ method to carefully peel off the perovskite films (Figure 1b). With this approach, the morphology of the buried, i.e., the perovskite–SAM interface can be imaged, in this case, using a combination of AFM and SEM. Representative AFM images of the top and bottom surfaces of perovskite films are presented in Figure 1c,d. From these, it is evident that the top surface is remarkably flat and homogeneous (RMS roughness ∼20 nm); however, in stark contrast, the perovskite surface from the buried interface has an irregular structure, exhibiting a large roughness (RMS roughness ∼30 nm) and the presence of numerous voids, the depth of which was determined to be in the region of 80 nm—20% of the overall perovskite film thickness.

We subsequently conducted a detailed investigation into the influence of film thickness on the genesis and dimensions of the voids present at the buried interface. To achieve this, the perovskite film thickness was modified by modulating the concentration of precursor solutions. The resulting SEM images obtained over the concentration range of 1.0 to 1.8 mol dm^–3^ are shown in Figure 1e. Over this range, the mean film thickness increased from 300 to 800 nm, and from the SEM images, it can be observed that there is an accompanying increase in the number and size of voids at the buried interface. The relationship between concentration and measured film thickness is given in Figure S1**.** The photographs shown in the inset also highlight that when the films are viewed from the backside, i.e., looking at the buried interface, the film cloudiness/opacity increases with increasing thickness. Based on these observations, we hypothesize that the presence of voids at the bottom contributes to optical scattering, thereby rendering the bottom side of the perovskite films cloudy in appearance.

### Controlling Film Morphology at the Buried Interface

It is interesting that while comparable top surface morphologies are observed in films of all thicknesses investigated, the buried interface structure changes so profoundly. We hypothesize that this is a result of nonhomogeneous nucleation and subsequent grain growth of our additive-free perovskite films,^35^ where disparities in nucleation and growth dynamics contribute to the formation of voids, and while concentration impacts the early stages of film formation, films can recover during growth and thus present comparable top surface morphologies.

**Figure 2:**
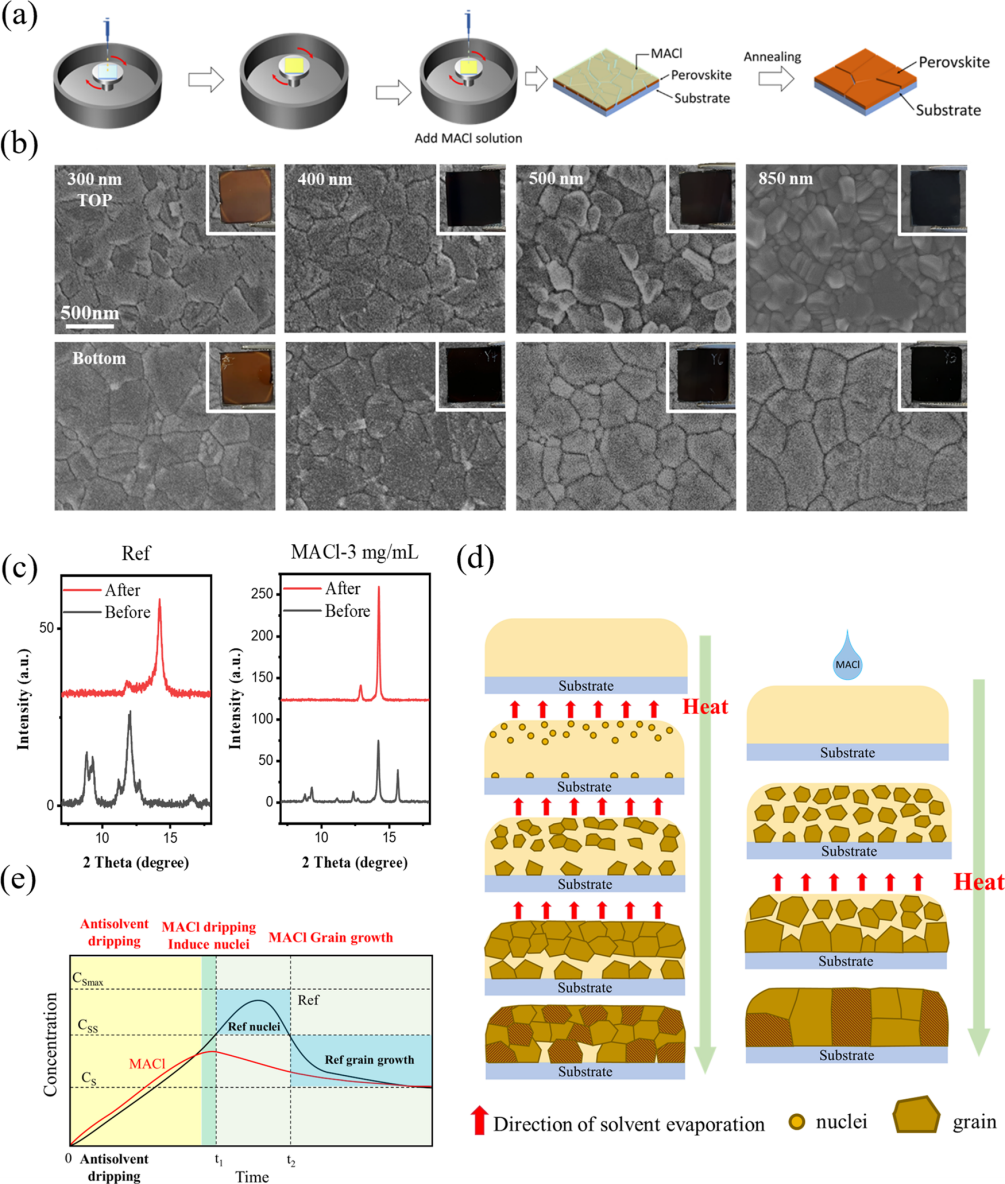
(a) Illustration showing the sequential deposition process for perovskite film fabrication. (b) Top- and bottom-view SEM images of perovskite films after sequential MACl deposition, with thickness given in annotation. (c) XRD patterns of perovskite films with and without sequential MACl deposition, displaying changes before and after the annealing process. (d) Schematics showing the proposed differences in film nucleation and growth. (e) LaMer diagrams for the untreated and sequentially treated perovskite films.

To eliminate void formation at the buried interface, we employed a sequential deposition method, utilizing MACl as an additive, to induce the formation of nuclei prior to thermal annealing^10^ (Figure 2a). We first explored the role of MACl concentration by introducing MACl dissolved in isopropanol over the concentration range of 1–7 mg/mL to perovskite layers with a thickness of ∼500 nm. SEM images of the resulting films showing the top surface and buried interface are shown in Figures S2 and S3. At both surfaces, continuous films that exhibit a monotonic increase in grain size are observed with increasing MACl concentration. The voids previously detected at the buried interface are again seen in the reference (MACl-free) films and are present, albeit in reduced quantity, in the 1 mg/mL sample and are fully eliminated at MACl concentrations >3 mg/mL. We also observed a subtle blue shift of the bandgap with increasing MACl concentration (Figure S4). We subsequently prepared PV devices (the Quantifying PV Device Performance Following Void Elimination section) over the same MACl concentration range with a fixed active layer thickness (500 nm). Based on the measured device performance metrics, a MACl concentration of 3 mg/mL was identified as optimum (Table S1).

Having optimized the MACl treatment conditions, we returned to varying the perovskite film thickness, depositing films over the range of 300–800 nm and treating these with 3 mg/mL of MACl. The resulting SEM images of the films are shown in Figure 2b. In addition to a subtle increase in grain size observed with increasing film thickness, the voids previously observed at the buried interface are completely absent across the entire thickness range. Visual analysis of the films, shown as photographs in Figure 2b, reveals a mirror-like appearance of the top and buried surfaces when the MACl treatment is carried out.

We then carried out X-ray diffraction (XRD) before and after thermal annealing to better understand changes in the microstructure. The full XRD patterns are shown in Figure S5, while Figure 2c shows the 5°–20° 2θ range. In the reference films, peaks associated with the photoinactive phase, δ-FAPbI_3_, are seen prior to annealing (11.8° 2θ), with some evidence of α-FAPbI_3_ formation following annealing (14.2° 2θ). Strikingly, when the sequential MACl treatment was employed, the nonannealed films visually appeared red/orange in color (Figure S6), and the XRD patterns showed the presence of α-FAPbI_3_ (14.2° 2θ) and δ-FAPbI_3_ (11.8° 2θ). Combining these observations may indicate the presence of α–δ junction regions in the films^36^ that on thermal annealing change color to a deep black with a mirror-like appearance, and they conclusively demonstrate the formation of α-FAPbI_3_ regions prior to thermal annealing when our MACl treatment is used.

We propose that these crystalline α-FAPbI_3_ regions play a significant role in the elimination of voids at the buried interface. We rely on LaMer theory to offer a more comprehensive explanation of the generation and subsequent elimination of these voids; the proposed mechanistic diagrams are outlined in Figure 2d,e. We suggest that the crystallization mechanism of the reference sample is heterogeneous. After solvent washing, the precursor solution concentration approaches the critical supersaturation point (C_SS_), and when placed on a hot plate, nuclei uniformly form at the film–atmosphere interface and at the buried film–substrate interface. Solvent evaporation at the upper interface will drive grain growth and coalescence, with the film growth direction being toward the substrate. Eventually, as the solvent gets depleted, the growing film can no longer structurally accommodate the spatial mismatch between the upper region of the film and the grains nucleated at the buried interface, leading to the formation of the observed voids.

This process is illustrated in Figure 2d. This phenomenon becomes more pronounced as the film thickness increases as thinner films experience shorter compression times during nuclei growth and are less affected by solute depletion, reducing the likelihood of pinhole and void formation. In contrast, thicker films experience higher compression probabilities due to increased thickness, making them more prone to pinhole and void formation. This can be correlated with the presence of voids at the bottom of films of different thicknesses in SEM images in Figure 1e. With the films prepared by our sequential deposition method, nuclei are already formed within the film without the need for thermal annealing, as confirmed by XRD (Figure 2c). Therefore, the crystallization process transforms from homogeneous crystallization to nonhomogeneous crystallization (Figure 2e), whereby the introduction of MACl (t1) triggers nucleation and crystallization followed by crystal growth with extended crystallization time, resulting in dense and large-sized grains through Ostwald ripening, shown schematically in Figure 2d. As shown in Figure S7, the introduction of MACl into the perovskite film significantly enhances the photoluminescence (PL) emission, with an approximately 6-fold increase in intensity. This enhancement indicates improved perovskite quality and reduced nonradiative recombination. The hypsochromic shift in the PL emission can be attributed to the incorporation of MA cations.

Additionally, the PL decay dynamics measured using time-correlated single-photon counting (TCSPC) reveal longer lifetimes for both recombination pathways in MACl-treated films. The fast decay component, τ_1_, associated with trap-assisted recombination, increases from 37 to 71 ns. Similarly, the slow decay component, τ_2_, corresponding to bimolecular recombination, also extends from 414 to 2306 ns. Overall, these observations support our conclusions that the MACl treatment results in a significant reduction of defect states.

### Quantifying PV Device Performance Following Void Elimination

**Figure 3:**
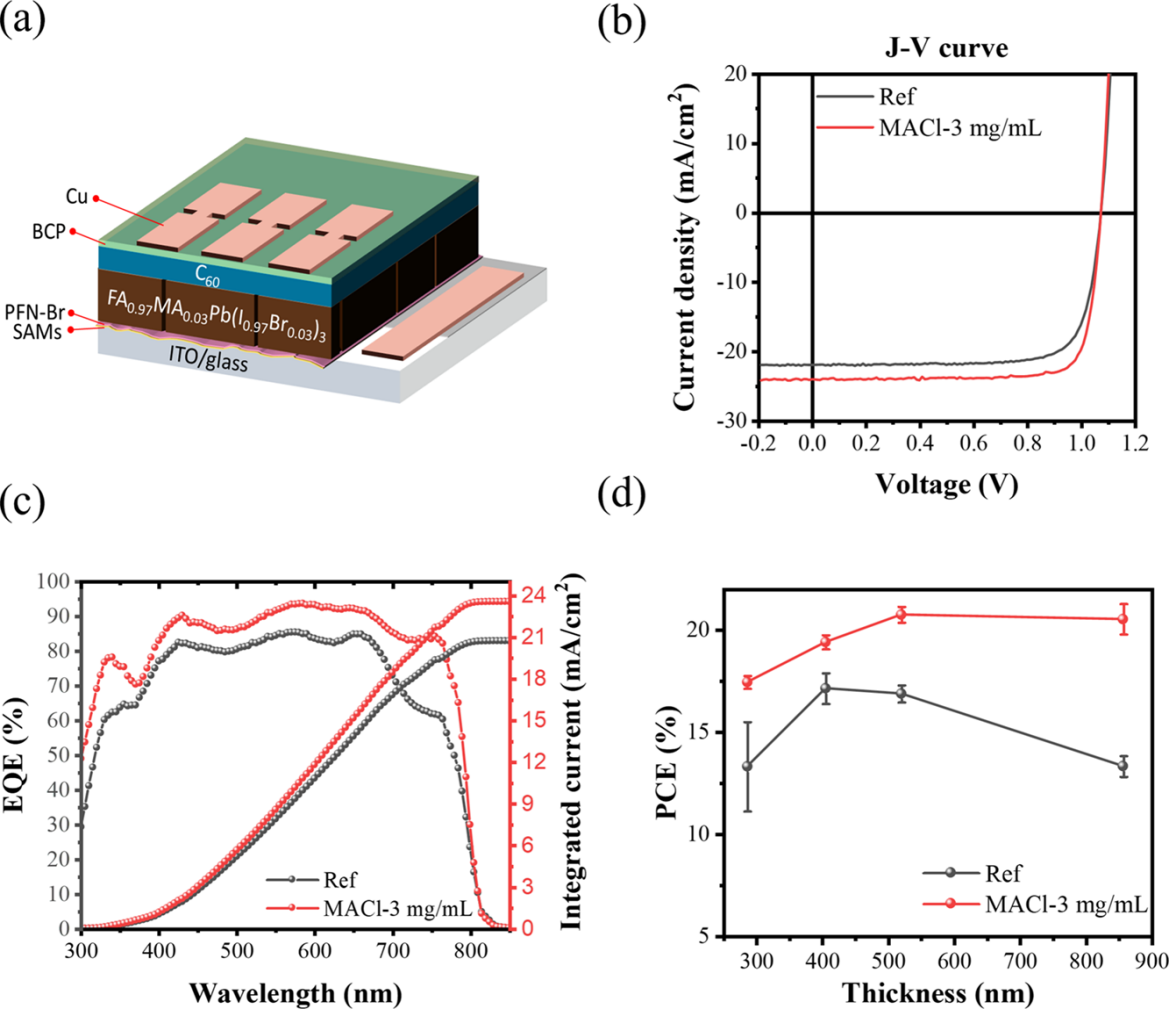
(a) Illustration of the p-i-n solar cell architecture. For the untreated (reference) and 3 mg/mL MACl-treated films, we show (b) representative *J*–*V* curves, (c) EQE and integrated short-circuit current density (*J*_SC_), and (d) statistical PCE data for active layer thicknesses from 300 to 850 nm.

To investigate the impact of eliminating the buried interface voids, we prepared PVs using FA_0.97_MA_0.03_Pb(I_0.97_Br_0.03_)_3_ films treated with MACl and compared with untreated films. All devices were of the p-i-n architecture with the general structure of ITO/SAM/PFN-Br/perovskite/C_60_/BCP/Cu, as shown in Figure 3a. A statistical summary of the device performance is given in Table S1. From the forward and reverse *J*–*V* scan data (Figure S8), the reference devices are seen to exhibit significant hysteresis, the magnitude of which decreases with increased MACl concentration, becoming negligible when the MACl concentration >3 mg/mL. The champion PCE for our reference devices was 19.02% compared with a PCE of 21.86% for the champion MACl-treated device (3 mg/mL). Analysis of the *J*–*V* data for the best-performing cells reveals enhancements of *J*_SC_ and FF, as shown in Figure 3b, following MACl treatment. The improvements in *J*_SC_ are supported by the enhanced external quantum efficiency (EQE) measurements seen following MACl treatment (Figure 3c).

This method was also applied to devices with varying thicknesses of perovskite layers, as depicted in Figure 3d, with the MACl concentration fixed at the optimized concentration of 3 mg/mL. For the untreated devices, the highest PCE was observed in the device with an active layer thickness of 400 nm, with PCE dropping substantially as the film thickness increased. We would anticipate a gradual increase in PCE over this thickness range owing to greater photon absorption, eventually tailing off owing to a combination of increased grain boundary concentrations and surface coarsening;^37^ however, here, the sharp drop in PCE for film thicknesses >400 nm is attributed to the voids present at the buried interface as seen in Figure 1e. Notably, following the MACl treatment, the PCE of devices prepared from all active layer thicknesses was consistent with the removal of the microstructural defects at the buried interface. We also prepared films and devices of the same architecture from pure FAPbI_3_ (Figure S9), where a significant improvement in device performance was also observed. While stability was not the primary focus of our investigations, we have carried out some preliminary work to evaluate stability using XRD.^38^ As shown in Figure S10, the results show less structural degradation in films treated with MACl.

### Role of Voids at the Buried Interface and *J*_SC_ Losses

**Figure 4:**
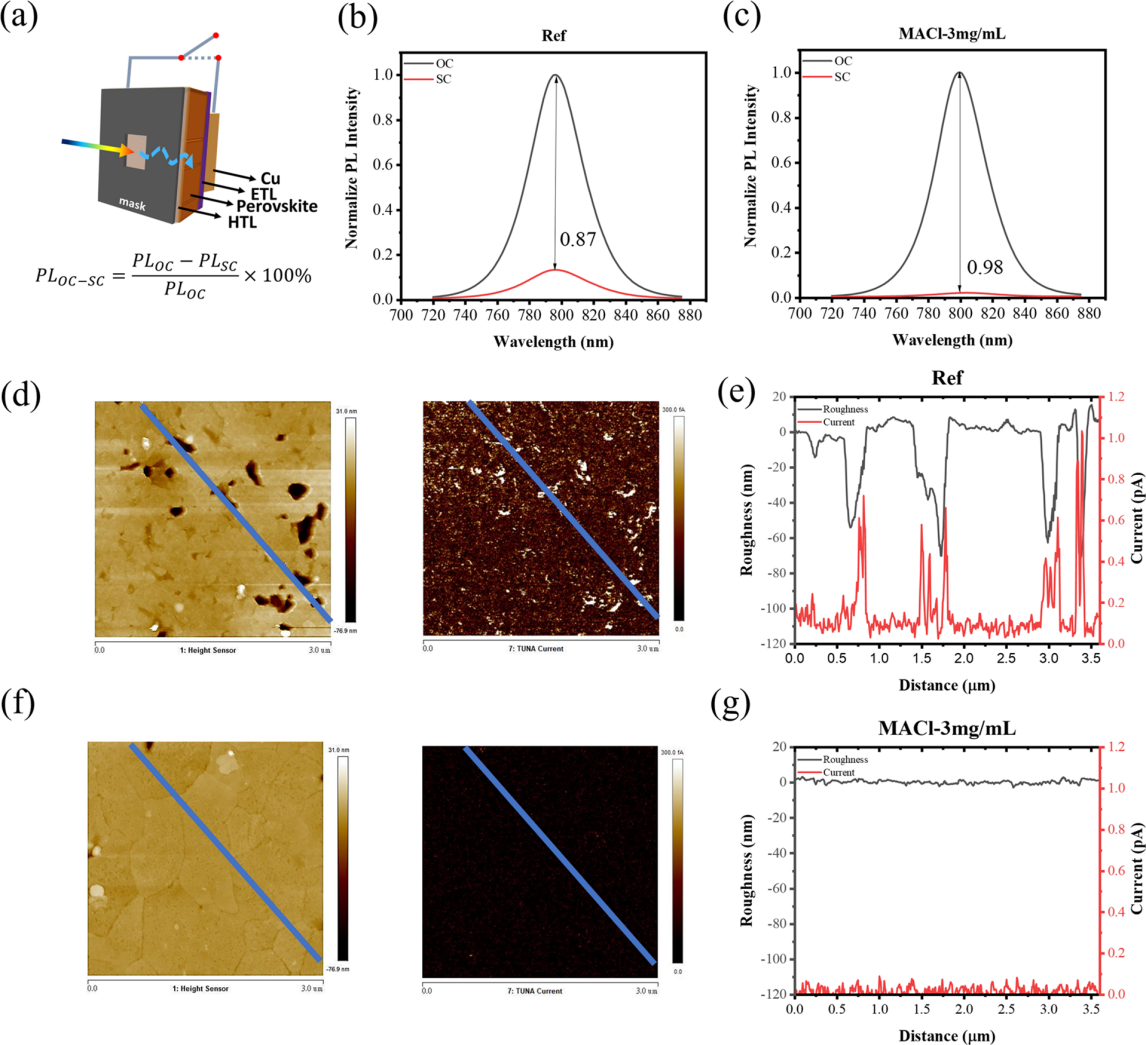
(a) Schematic showing the device PL measurement architecture. Measured PL quenching of the devices (b) untreated and (c) with sequential MACl treatment. C-AFM analysis of perovskite half-cells: (d) topography image showing the buried interface of untreated perovskites and (e) corresponding current and roughness profile. (f) Topography image showing the buried interface of MACl-treated perovskites and (g) corresponding current and roughness profile.

From our experimental data, we have observed significant *J*_SC_ losses in devices that are known to have active layers with a high void density at the HTL–perovskite interface. To better understand the origins of this, we employed device PL measurements to probe electron and hole extraction in untreated and MACl-treated films (Figure 4a). During measurement, free carriers in devices maintained at an open circuit will remain in the active layer and recombine; when this is by radiative pathways, the resultant PL can be monitored (PL_oc_). When the device is switched from open to short circuit, the external circuit can extract the charge carriers that were held in the active layer under open-circuit conditions. Charges trapped in the perovskite layer, i.e., those that cannot be extracted, can also generate a PL signal (PL_sc_), which can be correlated with the observed *J*_sc_ loss relating to inefficient charge extraction.^21,39^ Therefore, a larger difference in PL signals (PL_oc-sc_) between open-circuit and short-circuit states correlates with improved charge extraction, and this can be quantified using eq 1.
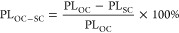
1

The results of device PL measurements for reference devices and those treated with 3 mg/mL of MACl are presented in Figure 4b,c, respectively. The untreated devices show PL quenching of 87% moving from open-circuit to short-circuit conditions; i.e., 87% of the free carriers are extracted to the external circuit. In contrast, devices that have undergone our MACl treatment show PL quenching of 98%. This is consistent with the differences in the EQE measured between untreated and treated devices (Figure 3c), where the integrated areas under the curves differ by 14%.

The enhanced charge extraction capability, reflected by the improved PL quenching efficiency, results from the reduced density of defects at the buried interface. When the device is held under short-circuit conditions under illumination, a high void density at the buried interface impedes photogenerated charges; therefore, more recombination occurs. Our sequential treatment improves the interfacial quality, thus increasing the charge extraction capability.

To investigate this further, we have used C-AFM to analyze the buried interface of perovskite films removed using the epoxy peel-off method; these measurements were made under dark conditions to assess the impact of voids on charge distribution. The morphology and current spatial distributions for untreated and MACl-treated perovskite films are shown in Figure 4d,e. The spatial data are overlaid on the line plots shown in Figure 4f,g, where in Figure 4f, we note large currents measured in the void regions, i.e., the regions with high roughness, in stark contrast to the flatter regions. This suggests that significant accumulated charges build up in the void regions. Such accumulated charge presents a challenge for effective charge extraction, particularly when the perovskite lacks direct contact with the HTL as is the case at the voids. Considering also the device PL data (Figure 4b,c), we suggest that this provides direct evidence of localized radiative recombination regions at and around the voids that impede effective carrier extraction and have a detrimental impact on *J*_sc_, thus the overall PCE.

### Extending to Other HTLs

**Figure 5:**
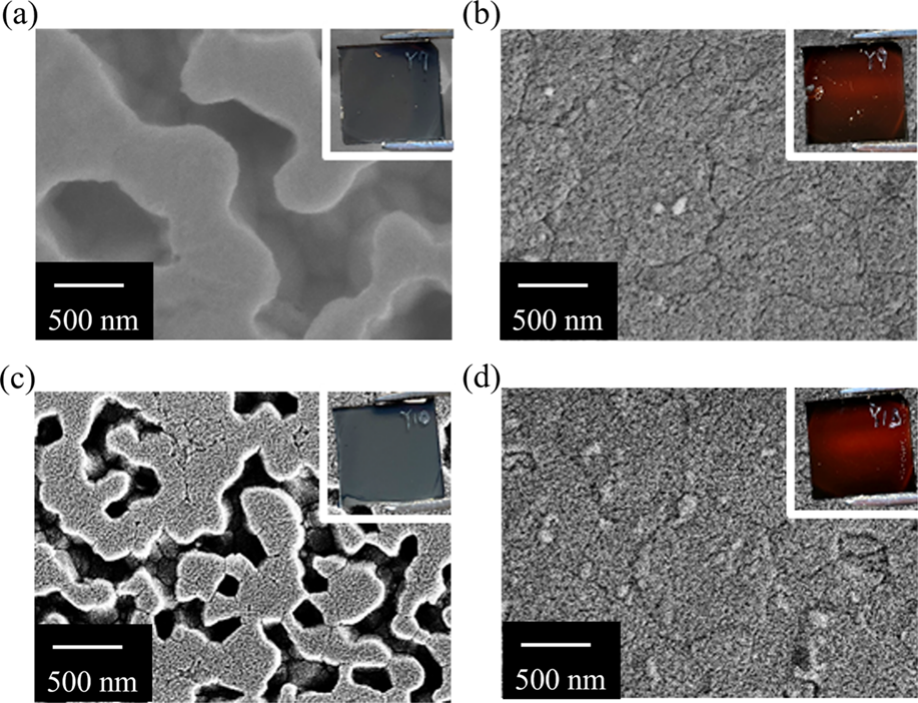
SEM images of the buried interfaces in perovskite layers: (a) PTAA substrate without MACl, (b) PTAA substrate following sequential MACl treatment, (c) PTPD substrate without MACl, and (d) PTPD substrate after sequential MACl treatment. The insets show photographs of the prepared films.

To investigate the applicability of our treatment to a wider range of device compositions and architectures, we explored the suitability of a range of common polymeric HTLs, specifically poly[bis(4-phenyl)(2,4,6-trimethylphenyl)amine (PTAA) and poly[*N*,*N*′-bis(4-butylphenyl)-*N*,*N*′-bisphenylbenzidine] (PTPD). The measured contact angles for both HTLs are shown in Figure S11, indicating their hydrophobic nature. SEM images showing the resulting buried interface of perovskite films deposited on each HTL are displayed in Figure 5, and in both cases, the untreated films show a substantial density of voids that percolate across the entire films. Previous research from Bi et al. has reported that increased hydrophobicity in HTLs results in fewer nuclei being formed at the buried interface leading to the growth of larger grains.^40^ Therefore, we hypothesize that with fewer bottom nuclei, the influence of solute depletion becomes more pronounced, resulting in the formation of more voids during crystallization. In contrast, the SEM images showing the buried interface of films deposited on the same HTLs, but subject to our MACl treatment (Figure 5b,d), show an appreciable reduction in void density.

### Widening the Perovskite Processing Window

Figure S12 presents the *J–V* characteristics for PSCs with PTAA and PTPD substrates, both exhibiting morphologies free of voids at the buried interface. These devices achieve PCEs > 19%—with an FF of 81.6 for the PTPD-based device and 84.1 for the PTAA-based device. This is consistent with the existing literature, which suggests that polymeric HTLs typically yield slightly lower PCEs in comparison to SAMs, largely due to the higher parasitic absorption associated with polymeric HTLs^33,34^

**Figure 6:**
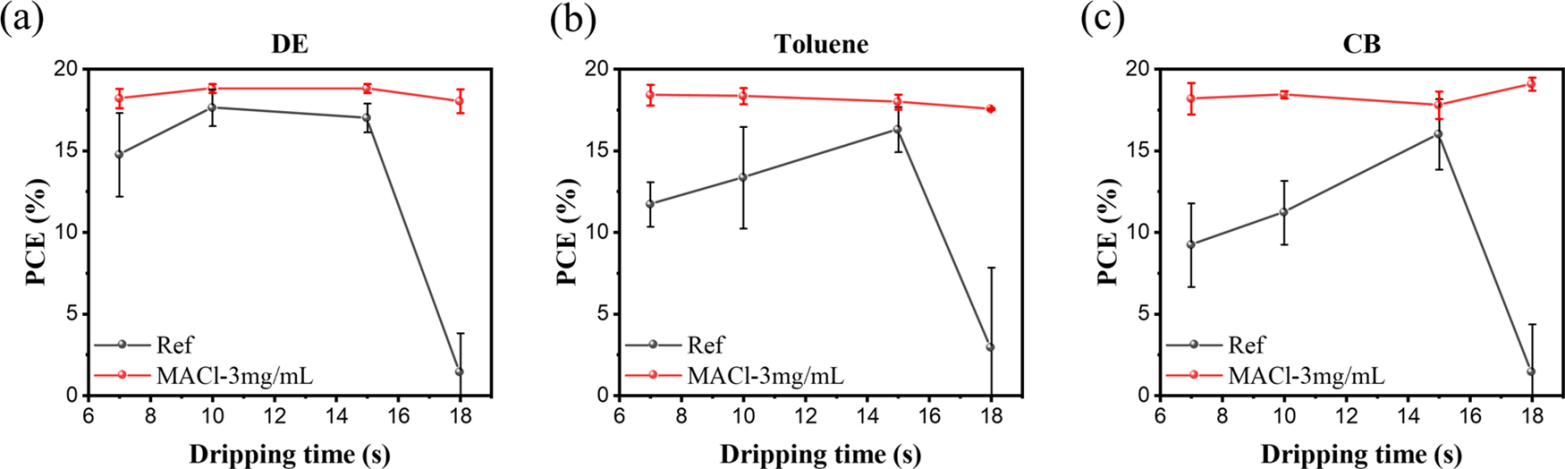
Performance comparison of untreated and MACl-treated PSCs. PCE data for cells, using (a) diethyl ether, (b) toluene, and (c) chlorobenzene as antisolvents, are plotted as a function of dripping time.

We have highlighted that our sequential MACl deposition process, when carried out following antisolvent dripping, can modify the crystallization mechanism of the films and significantly improve the morphology of the buried perovskite–HTL interface. This enhancement in the crystallization process creates perovskite films that are less sensitive to the antisolvent choice and also less sensitive to the time the antisolvent is introduced to the film. The data in Figure 6 show the measured PCE values for 400 nm-thick perovskite films deposited on PTAA as an HTL, untreated and also treated with MACl. We investigate three common antisolvents, namely, diethyl ether, toluene, and chlorobenzene, and vary the antisolvent dripping time from 7 to 18 s after spinning. In the untreated devices, device performance is highly sensitive to antisolvent choice and dripping time (Figure 6a–c). When the antisolvent is added too early, the precursor solution fails to reach near-supersaturation, resulting in uneven growth of nuclei at the top surface of the films. Consequently, after crystallization, voids form not only at the bottom but also at the top of the film. Conversely, adding the solvent too late, i.e., when the precursor solution has already reached a state of supersaturation, leads to the formation of nonuniform nucleation. Here, the devices prepared following the MACl treatment exhibit insensitivity to both the nature of the antisolvent and the dripping time; we attribute this to the added MACl initiating nucleation and subsequent crystallization (Figure 2), rendering the process of crystallization to be less sensitive solution concentration as it approaches the critical supersaturation point controlled by the antisolvent dripping process.^41^

## Conclusions

The presence of voids at the buried interface in FA-based PSCs has a significant, negative impact on device performance. Here, we developed and deployed a novel MACl treatment that eliminates the presence of such defects. We have studied the perovskite film morphology at the buried interface, using a recently developed delamination technique to gain access to this interface, using a combination of SEM, C-AFM, and steady-state and transient PL measurements to show that these voids lead to the accumulation of charges, diminishing extraction capabilities and consequently reducing PCE. This is highlighted in the PV device analysis when comparing performance values for devices with and without our MACl treatment. Here, we observed enhancements in both short-circuit current and fill factor after void repair, from 22.83 to 24.10 mA/cm^2^ and from 77.80 to 83.87%, respectively—resulting in champion PCEs of 19.02% (untreated) and 21.86% (MACl-treated). The MACl treatment is applicable to a range of commonly utilized HTLs, shown here using SAMs and polymeric p-type examples, and independent of perovskite active layer thickness, demonstrating the applicability of our method. Additionally, the introduction of MACl treatment widens the process window for PSCs, reducing the sensitivity of this critical processing step. Overall, the combined results provide a convenient path for the reproducible preparation of PSCs by using a range of common HTLs with active layer thicknesses that span PV and other emerging applications.

## Experimental Section

### Device Fabrication

Perovskite solutions with the composition (FAPbI_3_)_0.97_(MAPbBr_3_)_0.03_ were prepared by mixing FAPbI_3_ (NH_2_CHNH_2_PbI_3_) and MAPbBr_3_ (CH_3_NH_3_PbBr_3_) in a volume ratio of 97:3. The FAPbI_3_ precursor solution was prepared by dissolving an equal molar ratio of 1.5 M formamidinium iodide (FAI, > 99.99%, GreatCell Solar) and lead iodide (PbI_2_, 99.99%, TCI) in a solvent mixture of *N,N*-dimethylformamide (DMF) (anhydrous, 99.8%, Sigma-Aldrich) and *N*-methyl-2-pyrrolidone (NMP) (≥99%, Merck) (7.5:2.5 in volume ratio). The MAPbBr_3_ precursor solution was prepared by dissolving an equal molar ratio of 1.5 M methylammonium bromide (MABr, > 99.99%, GreatCell Solar) and lead bromide (PbBr_2_, 99.99%, TCI) in a solvent mixture of DMF and NMP (7.5:2.5 in volume ratio). The FAPbI_3_ solution was stirred at 60 °C, whereas the MAPbBr_3_ solution was at room temperature for 1 h to dissolve the precursor materials. PTAA (Mw 33,000, PID 2.7) and polyTPD were purchased from Ossila Ltd. and used as purchased. MeO-2PACz was purchased from TCI. Poly(9,9-bis(3′-(*N,N-*dimethyl)-*N*-ethylammoinium-propyl-2,7-fluorene)-*alt*-2,7-(9,9-dioctylfluorene))dibromide (PFN-Br) was purchased from 1-Material.

Inverted planar perovskite devices were prepared and fabricated as ITO/PTAA/PFN-Br/perovskite/C60/BCP/Cu. ITO-coated glass was sequentially cleaned with soap, deionized water, acetone, and isopropanol in an ultrasonic bath, followed by drying under a nitrogen stream, and finally treated with oxygen plasma. The HTL, including PTAA (2.5 mg/mL in toluene), PTPD (2.5 mg/mL in toluene), or MeO-2PACz, was pin-coated directly onto the cleaned substrate. PFN-Br (0.00625 wt % in methanol) was filtered with 0.45 μm PTFE and then spin-coated at 5000 rpm for 20 s. Perovskite layers were deposited using the antisolvent dripping method with post-treatment. Specifically, the precursor solutions were heated to 60 °C and deposited by spin coating at 4000 rpm for 20 s while dripping diethyl ether at the 10th second. After the diethyl ether dripping step, 15 μL of MACl solution, with concentrations varying from 3 to 7 mg/mL in isopropanol, was dynamically coated onto the film surface at 4000 rpm for 30 s. The perovskite films were formed by annealing at 60 °C for 1 min and 100 °C for 60 min. C60 and bathocuproine (BCP) were thermally evaporated on the substrates. Eventually, the devices were completed by the thermal evaporation of 100 nm of Cu.

### Characterization

Device *J*–*V* characteristics were measured with a Keithley 2400 source meter. The devices were illuminated by an AM 1.5 xenon lamp solar simulator (Enlitech) at 1 sun equivalent intensity, calibrated with a Si photodiode. EQE spectra were measured by the Bentham PVE300 photovoltaic QE system. Top-view and cross-section SEM images were obtained by a LEO Gemini 1525 field emission gun scanning electron microscope (FEG SEM). The accelerating voltages ranged from 3 to 5 kV, and working distances ranged from 3 to 5 mm. Films were coated with a thin (<10 nm) metal (Au/Cr) layer to facilitate imaging. Steady-state PL spectra and TCSPC were measured by an FS5 spectrofluorometer (Edinburgh). Ultraviolet–visible (UV–vis) spectra were measured by a Shimadzu UV-2600 UV–vis spectrometer. XRD data were obtained using a Bruker D2 PHASER instrument operating at 40 kV and 40 mA. C-AFM measurements were conducted using a Bruker Dimension Icon system equipped with ScanAsyst mode. During the C-AFM scans, a bias voltage of 200 mV was applied to the sample, enabling the measurement and mapping of current on the film surface.

## Supporting Information Available

Correlation between perovskite concentration and thickness; SEM of top view and buried interfaces of perovskite films; UV–vis spectra and Tauc plots; XRD of MACl-treated perovskites; photographs of unannealed perovskite films; PL and TCSPC of perovskites films; *J*–*V* curves and hysteresis index; UV–vis of FAPbI_3_ films and *J*–*V* characteristics of FAPbI_3_ perovskite devices; XRD of aged perovskite films; contact angle on PTAA/PTPD; and *J*–*V* curves of PTAA/PTPD devices (PDF)
