# Supplementary material for: Overcoming Microstructural Defects at the Buried Interface of Formamidinium-Based Perovskite Solar Cells
Source: ACS Appl Mater Interfaces. 2024 Aug 27;16(36):47763–72. doi: 10.1021/acsami.4c11052 (PMC11403615; doi:10.1021/acsami.4c11052)
Supplement: Supplementary file 1 — am4c11052_si_001.pdf [file am4c11052_si_001.pdf]

Supporting Information

Overcoming microstructural defects at the buried interface of formamidinium-based perovskite solar cells

Heng-Yi Lin[a], Zhongyao Jiang[b], Shi-Chun Liu[a], Zhaoyi Du[b], Shih-En Hsu[a], Yun-Shan Li[a], Wei-Jia Qiu[a], Hongta Yang[a], Thomas J. Macdonald[c], Martyn A. McLachlan[b] and Chieh-Ting Lin[a, d*]*

a. Department of Chemical Engineering, National Chung Hsing University, 145 Xingda Road, Taichung 40227, Taiwan

b. Department of Materials, Molecular Sciences Research Hub, Imperial College London, 82 Wood Ln, W12 0BZ, U.K.

c. Department of Electronic & Electrical Engineering, University College London, London, Torrington Place, WC1E 7JE, U.K.

d. Innovation and Development Center of Sustainable Agriculture, National Chung Hsing University, 145 Xingda Road, Taichung 40227, Taiwan

* Corresponding authors' email:

martyn.mclachlan@imperial.ac.uk

c.lin15@nchu.edu.tw

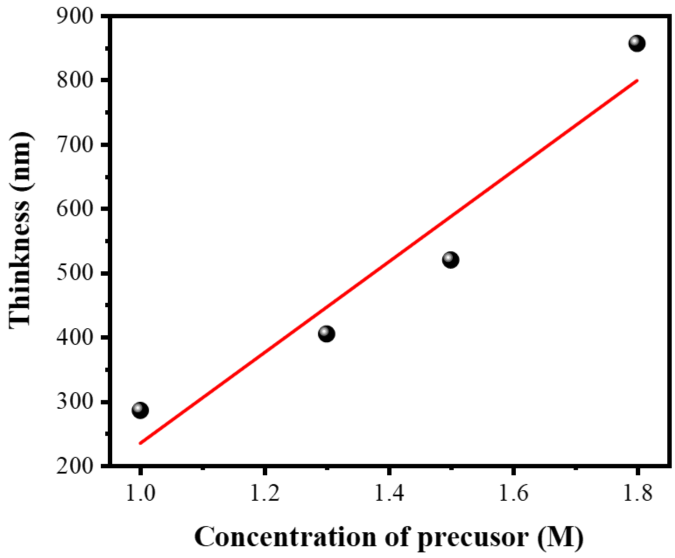

Figure S1. The correlation between perovskite precursor concentrations and the resultant film thickness.

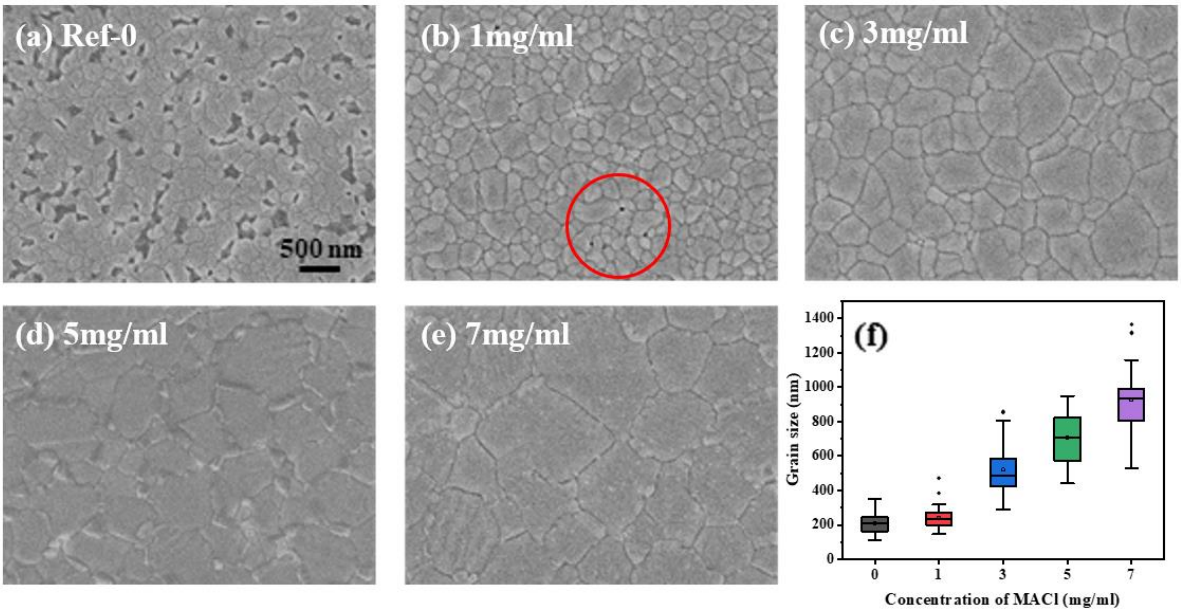

Figure S2. SEM images of the buried interface of perovskites treated with varying concentrations of MACl/IPA solutions: (a) reference (0 mg/ml), (b) 1 mg/ml, (c) 3 mg/ml, (d) 5 mg/ml, and (e) 7 mg/ml. (f) Grain size distribution of perovskite films as a function of MACl/IPA concentration.

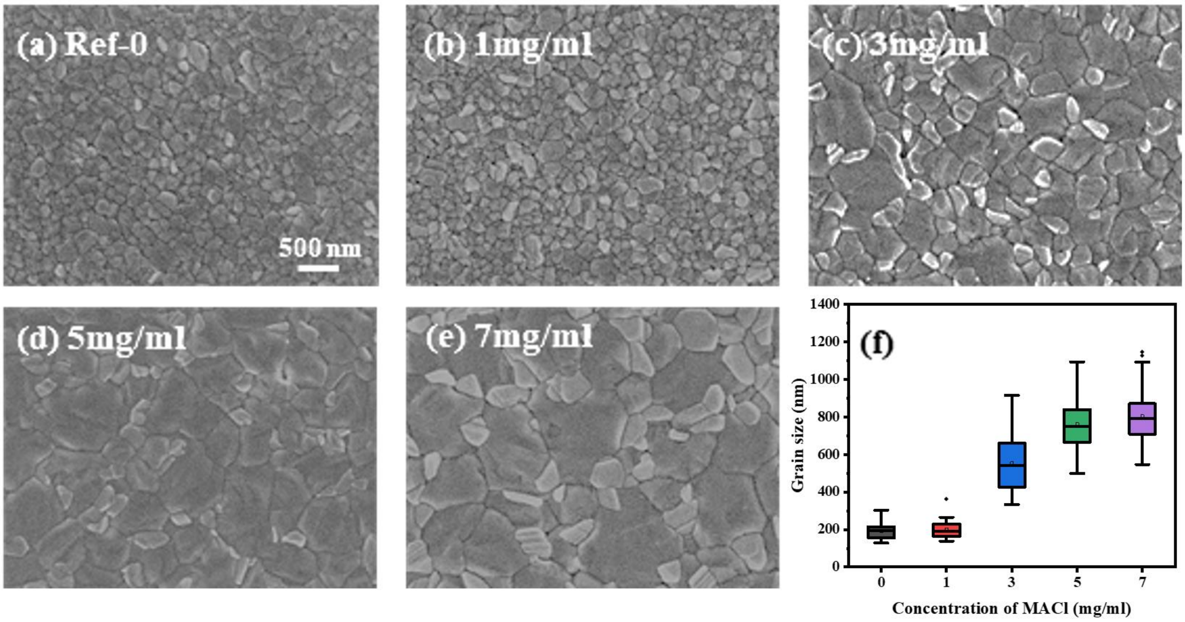

Figure S3. Top-view SEM images of perovskites treated with varying concentrations of MACl/IPA solutions: (a) reference (0 mg/ml), (b) 1 mg/ml, (c) 3 mg/ml, (d) 5 mg/ml, and (e) 7 mg/ml. (f) Grain size distribution of perovskite films as a function of MACl/IPA concentration.

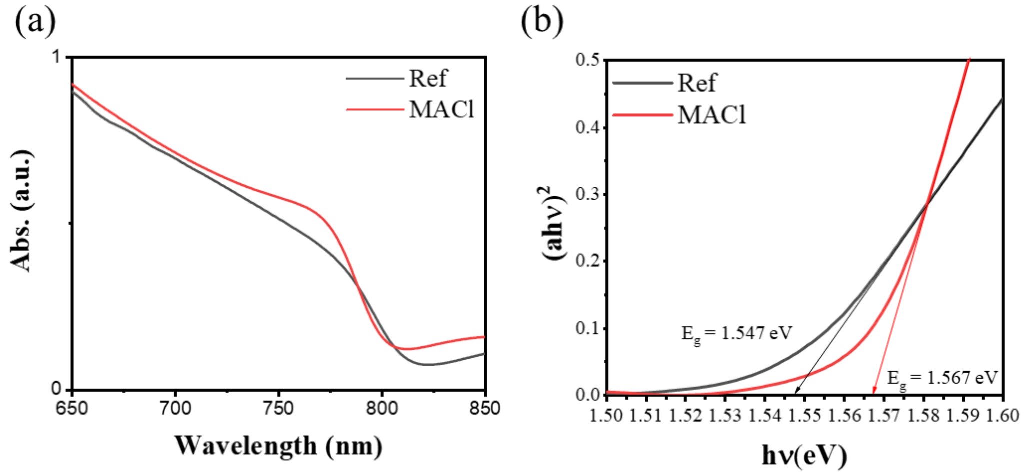

Figure S4. (a) UV-vis absorption spectra of perovskite films with and without MACl treatment. (b) Tauc plot calculated from the UV-vis spectra.

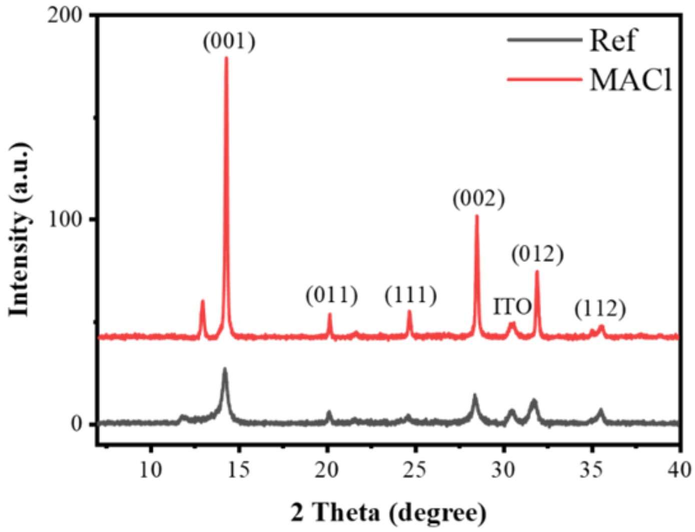

Figure S5. XRD of the $(FAPbI_3)_{0.97}(MAPbBr_3)_{0.03}$ perovskite with and without sequential MACl treatment.

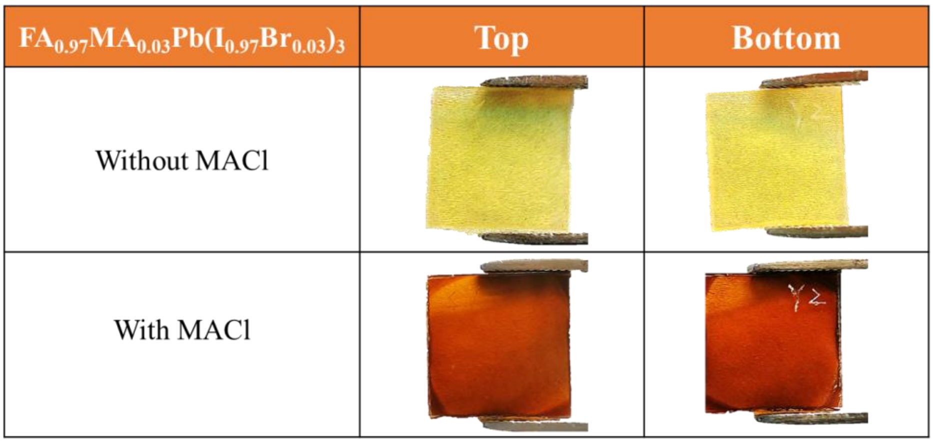

Figure S6. The pictures of the $(FAPbI_3)_{0.97}(MAPbBr_3)_{0.03}$ perovskite films with and without sequential MACl treatment (3mg/ml) before thermal annealing.

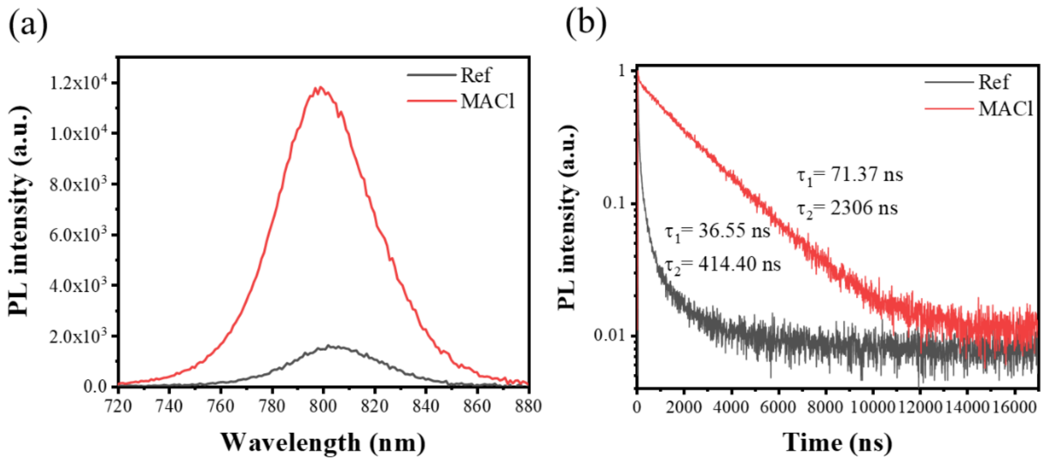

Figure S7. (a) Steady-State Photoluminescence and (b) Time-correlated single-photon counting (TCSPC) of the perovskite film with and without sequential MACl treatment (3mg/ml).

Table S1. Parameters of perovskite solar cell devices with and without sequential MACl-treatment. Here the number after the MACl represents the concentration of the MACl / IPA solution in mg/mL (MACL-1 means 1 mg/mL MACl / IPA).

**Condition**	***V*oc (V)**	***J*sc (mA/cm2)**	**FF (%)**	**PCE (%)**
**Ref**	1.06 ±0.02<br>(1.07)	22.79 ±0.32<br>(22.83)	76.30 ±1.18<br>(77.80)	18.86 ±0.50<br>(19.02)
**MACl-1**	1.07 ±0.02<br>(1.07)	22.20 ±0.55<br>(22.32)	79.25 ±2.18<br>(80.90)	18.97 ±0.35<br>(19.44)
**MACl-3**	1.08 ±0.01<br>(1.08)	23.81 ±0.35<br>(24.10)	82.59 ±0.81<br>(83.87)	21.17 ±0.45<br>(21.86)
**MACl-5**	1.08±0.01<br>(1.08)	23.59 ±0.23<br>(23.89)	81.58 ±0.96<br>(81.37)	20.77 ±0.19<br>(21.86)
**MACl-7**	1.09±0.01<br>(1.10)	23.69 ±0.36<br>(24.03)	80.97 ±2.18<br>(80.76)	20.89 ±0.40<br>(21.30)

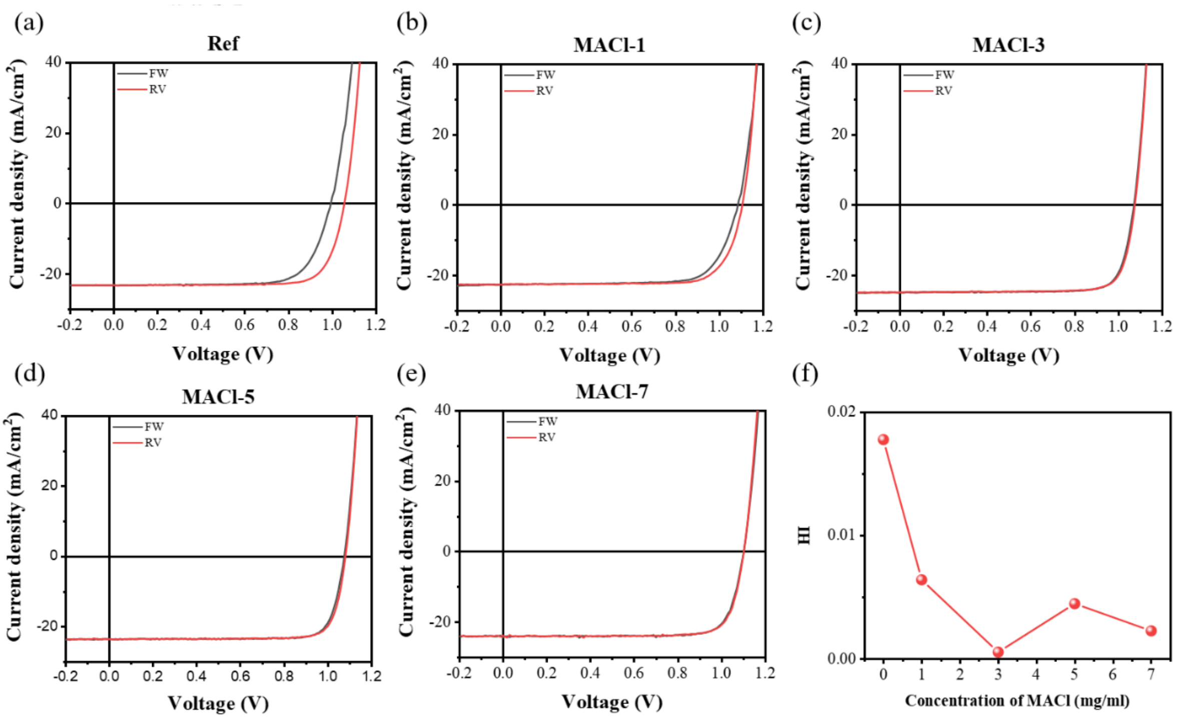

Figure S8. J-V curves of the devices without (a) and with (b-e) sequential deposition of MACl. Here the number after the MACl represents the concentration of the MACl / IPA solution in mg/mL (MACL-1 means 1 mg/mL MACl / IPA). (f) The hysteresis index of the device with various concentration of MACl.

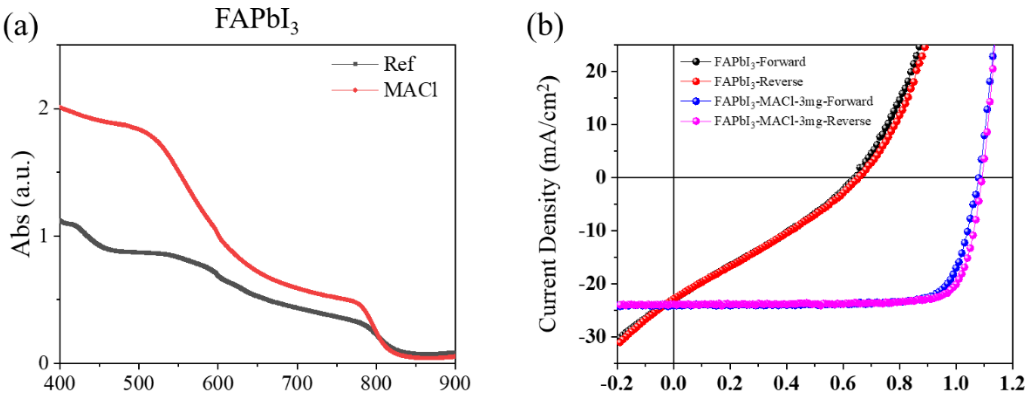

Figure S9(a) UV-Vis spectra of $FAPbI_3$ thin films with and without MACl treatment (b) J-V characteristics of devices employing $FAPbI_3$ as the absorber, with and without MACl treatment, the PCE increases from 4.2% in the untreated device to 21.0% following MACl treatment.

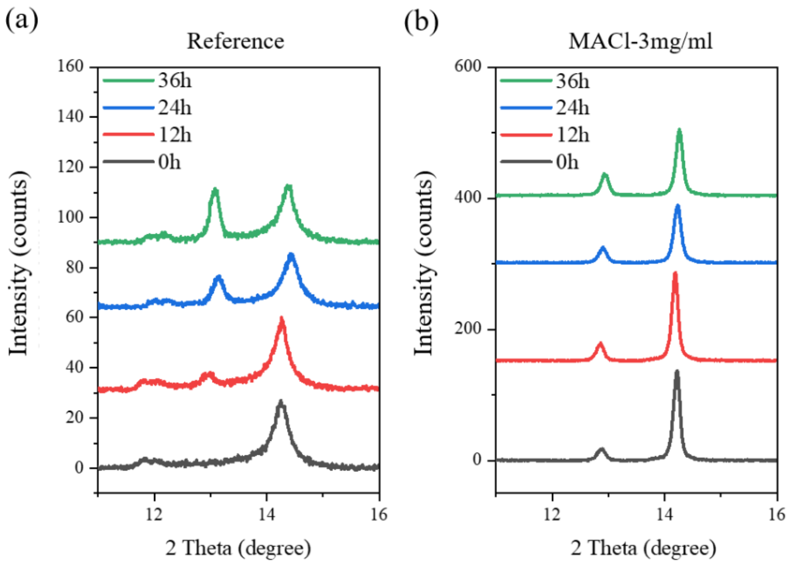

Figure S10. XRD patterns of (a) pristine perovskite film and (b) perovskite film treated with MACl, both aged in ambient air at 30% relative humidity for 36 hours.

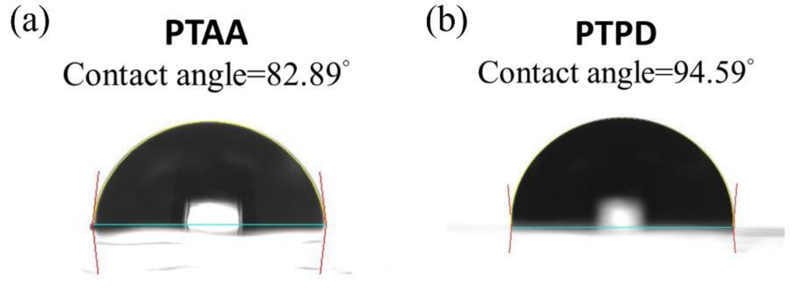

Figure S11. The contact angle measurement on (a) PTAA and (b) PTPD.

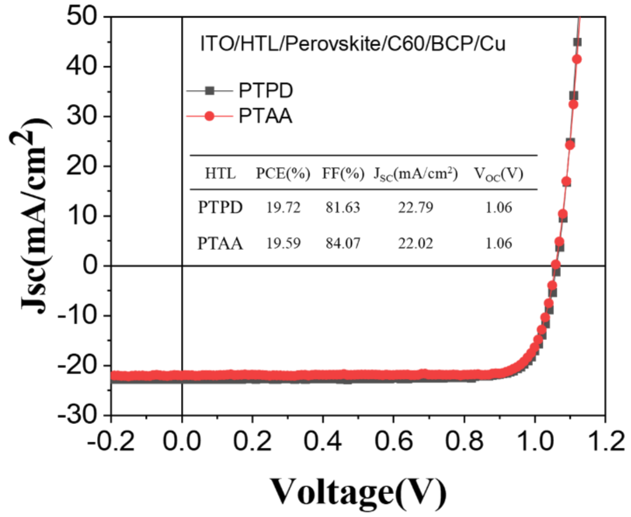

Figure S12. J-V curve of perovskite solar cells employing PTAA (Poly[bis(4-phenyl)(2,4,6-trimethylphenyl)amine]) and PTPD (Poly(triarylamine)) as hole transporting layers.
